# A diagnostic model based on bioinformatics and machine learning to differentiate bipolar disorder from schizophrenia and major depressive disorder

**DOI:** 10.1038/s41537-023-00417-1

**Published:** 2024-02-14

**Authors:** Jing Shen, Chenxu Xiao, Xiwen Qiao, Qichen Zhu, Hanfei Yan, Julong Pan, Yu Feng

**Affiliations:** 1 The Affiliated Jiangsu Shengze Hospital of Nanjing Medical University, 251221 Suzhou, China; 2 The Fourth People’s Hospital of Wujiang District, 215231 Suzhou, China; 3 The University of New South Wales, 2052 Sydney, Australia; 4 The University of Melbourne, 3010 Melbourne, Australia

**Keywords:** Emotion, Human behaviour

## Abstract

Bipolar disorder (BD) showed the highest suicide rate of all psychiatric disorders, and its underlying causative genes and effective treatments remain unclear. During diagnosis, BD is often confused with schizophrenia (SC) and major depressive disorder (MDD), due to which patients may receive inadequate or inappropriate treatment, which is detrimental to their prognosis. This study aims to establish a diagnostic model to distinguish BD from SC and MDD in multiple public datasets through bioinformatics and machine learning and to provide new ideas for diagnosing BD in the future. Three brain tissue datasets containing BD, SC, and MDD were chosen from the Gene Expression Omnibus database (GEO), and two peripheral blood datasets were selected for validation. Linear Models for Microarray Data (Limma) analysis was carried out to identify differentially expressed genes (DEGs). Functional enrichment analysis and machine learning were utilized to identify. Least absolute shrinkage and selection operator (LASSO) regression was employed for identifying candidate immune-associated central genes, constructing protein-protein interaction networks (PPI), building artificial neural networks (ANN) for validation, and plotting receiver operating characteristic curve (ROC curve) for differentiating BD from SC and MDD and creating immune cell infiltration to study immune cell dysregulation in the three diseases. RBM10 was obtained as a candidate gene to distinguish BD from SC. Five candidate genes (LYPD1, HMBS, HEBP2, SETD3, and ECM2) were obtained to distinguish BD from MDD. The validation was performed by ANN, and ROC curves were plotted for diagnostic value assessment. The outcomes exhibited the prediction model to have a promising diagnostic value. In the immune infiltration analysis, Naive B, Resting NK, and Activated Mast Cells were found to be substantially different between BD and SC. Naive B and Memory B cells were prominently variant between BD and MDD. In this study, RBM10 was found as a candidate gene to distinguish BD from SC; LYPD1, HMBS, HEBP2, SETD3, and ECM2 serve as five candidate genes to distinguish BD from MDD. The results obtained from the ANN network showed that these candidate genes could perfectly distinguish BD from SC and MDD (76.923% and 81.538%, respectively).

## Introduction

Bipolar disorder (BD) is a disorder of recurrent episodes of hyperthymia and depression that negatively affects the lives of most patients despite its association with creativity^1^, and patients with BD have the highest suicide rate of all psychiatric disorders, about 20–30 times that of the general population^2^. However, correctly diagnosing BD is a very difficult task, and in related studies, it was observed that more than half of the doctors were unable to correctly diagnose BD^3^. These misdiagnoses may result in a cascade of negative outcomes, and patients may receive inadequate or inappropriate treatments, which fail to alleviate the symptoms or damage of the disease and may even cause further deterioration of their mood^4^.

In the diagnosis of BD, it is most easily confused with schizophrenia (SC) and major depressive disorder (MDD). SC is a neuropsychiatric disorder that usually occurs in adolescents or young adults and mostly lasts throughout one’s entire lifespan. It is typically characterized by hallucinations, delusions, apathy, and social withdrawal. However, patients with BD also present with symptoms similar to those of schizophrenics^5^, and genome-wide association studies have shown a substantial genetic overlap between SC and BD^6^, making it extraordinarily difficult to distinguish between the two disorders. When patients with BD have major depressive episodes, their clinical phenotype is not fundamentally different from those with MDD (monophasic depression). Therefore, many patients with BD are frequently misdiagnosed as having MDD (monophasic depression)^7^.

Thus, distinguishing BD from SC and MDD has become one of the main tasks in efficiently diagnosing the disorder. This study aims to establish a diagnostic model to distinguish BD from SC and MDD in multiple public datasets using bioinformatics and machine learning and to provide new ideas for diagnosing BD in the future.

This study used different datasets, including different sample sources, experimental conditions, and data from different time points. This diversity helps to increase the comprehensiveness and reliability of research. In addition, various types of datasets can help us verify the reliability of diagnostic models.

In addition, the bioinformatics and machine learning methods used in this study are capable of processing large-scale data, mining hidden patterns, identifying complex interactions, etc., which have implications for diagnosing and distinguishing diseases.

## Methodology

### Materials

The brain tissue datasets GSE92538-GPL10526, GSE92538-GPL17027, and GSE12654 were chosen using the GEO database (https://www.ncbi.nlm.nih.gov/geo/)^8^ as training groups, each containing patients with SC, BD, and MDD. The GSE18312 and GSE39653 datasets were chosen as the validation groups to distinguish BD from SC and MDD (both GSE18312 and GSE39653 were peripheral blood datasets), in order to increase the sample size for analysis and enhance the reliability of the study, we merged and removed batch effects from multiple datasets.

**Fig. 1:**
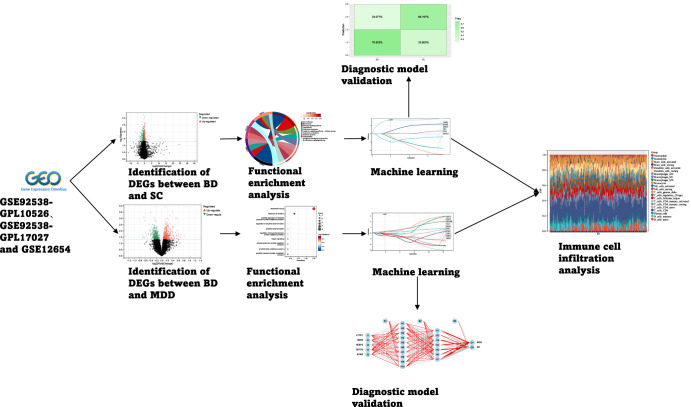
Flow chart.

To merge multiple datasets, the datasets were initially merged by employing the R package. This was followed by the removal of the batch effect function of the R package limma (version 3.42.2) for the purpose of eliminating the batch effect, after which the matrix was attained. Figure 1 shows the detailed process. Supplementary table 1 shows the detailed datasets information.

### Identification of differentially expressed genes

Linear Models for Microarray Data (Limma) analysis^9^ is a generalized linear model-based differential expression analytical technique. In this study, the R package limma (version 3.40.6) was employed for differential analysis to attain differentially expressed genes (DEGs) among variant comparison groups and controls. During this research, |log2 fold change (FC)| > 1 and *P* value < 0.05 were chosen as the criteria for identifying DEGs using the Limma package, and heat map and volcano plots of DEGs in the individual and combined datasets were visualized by sangerBox, respectively^10^.

The criteria of |log2 fold change (FC)| > 1 and *P* value < 0.05 are essential to ensure that the selected differentially expressed genes are relatively reliable. This helps reduce false-positive results and enhances the credibility of the research findings. |log2 fold change (FC)| > 1 signifies that the differential expression genes have a fold change of at least 2, which is typically considered biologically significant in the field of biology. A *P* value < 0.05 indicates statistical significance, making it easier to determine which genes exhibit significance in differential expression between diseases.

### Gene function enrichment analysis

The DEGs in the single and combined dataset obtained according to the above processes were cross-screened by the Venn diagram, and the genes capable of distinguishing the three diseases were obtained to further perform gene function enrichment analysis, in order to analyze the functional pathways in which these genes play a role. To conduct gene set functional enrichment analysis, the Kyoto Encyclopedia of Genes and Genomes (KEGG) rest application programming interface (API) (https://www.kegg.jp/kegg/rest/keggapi.html) was utilized for the purpose of obtaining the most recent KEGG Pathway gene annotations. The R package org.Hs.eg.db (version 3.1.0)^11^ in the GO annotation of genes was utilized as background. The genes were mapped to the background set, and enrichment analysis was carried out by employing the R package clusterProfiler (version 3.14.3)^12^ to obtain the gene set enrichment results. The minimum and maximum gene sets were defined at 5 and 5000, respectively, based on gene expression patterns and phenotypic categories, and a *P* value < 0.05 and an FDR < 0.1 were regarded as statistically significant.

### Machine learning identification for candidate genes to distinguish three diseases

LASSO is a regression approach for variable selection and regularization that enhances the predictive power and interpretability of a statistical model^13^. Survival time, survival status, and gene expression data were integrated for regression analysis by employing the lasso-cox technique utilizing the glmnet^14^ function in the R package. To identify the optimal model, a 10-fold cross-validation procedure was established.We use it to screen genes that can distinguish between SC and BD, as well as between BD and MDD.“We defined the outcome event as the clinical diagnosis of the disease, and we defined the survival event as fixed, in order to minimize its bias on the predictive model construction.”

### Construction of protein-protein interaction networks

The GeneMANIA database, a versatile and ergonomic website for the development of hypotheses regarding gene function, assessment of gene lists, and prioritization of genes for functional analysis, was employed to generate the protein-protein interaction network (PPI)^15^.

### Diagnostic model validation

ROC analysis was performed by employing the pROC function^16^ in the R package for the purpose of obtaining AUC, which was then evaluated along with confidence intervals (CI) utilizing the CI function of pROC to determine the final AUC findings, which were visualized using sangerBox. The signature gene expression was observed in the individual datasets versus the combined datasets and in the test groups (GSE18312 and GSE39653). In addition to this, neuralnet^17^ in the R package was employed for the construction of an artificial neural network (ANN) for the feature genes attained via the method described above, which led to the construction of a highly precise diagnostic model.

### Immunoinfiltration analysis

IOBR^18^ is a computational tool for immuno-oncology biology studies. Here the CIBERSORT^19^ method was chosen based on the expression profiles using the R package IOBR to calculate the 22 immune infiltrating cell scores for each sample. Immune cell infiltration analysis was conducted via CIBERSORT in the R package, and its correlation was calculated using the spearman coefficient, and a heat map of infiltrating immune cell correlation was carried out by employing the corrplot in the R package.Further, we will conduct correlation analysis between the 6 target genes and significantly different immune cells.

## Results

### Identification of DEGs between BD and SC

**Fig. 2:**
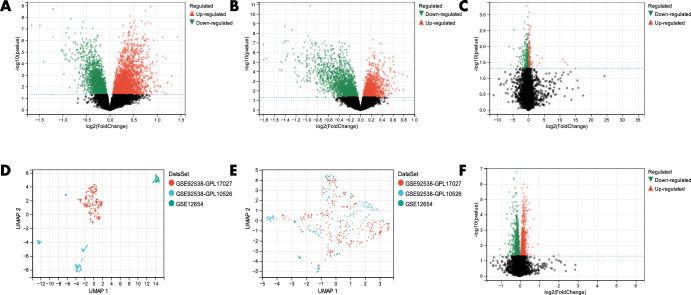
DEGs between BD and SC. **A** volcano plots of DEGs in dataset GSE92538-GPL10526; **B** volcano plots of DEGs in dataset GSE92538-GPL17027; **C** volcano plots of DEGs in dataset GSE12654; **D**, **E** UMAP plots before and after removal of batch effects; **F** combined volcano plots of DEGs in the dataset.

The Limma method allowed the identification of about 4655 DEGs in dataset GSE92538-GPL10526. Out of these, about 2620 were up-regulated, and 2035 were down-regulated (Fig. 2A). About 3297 DEGs were identified in dataset GSE92538-GPL17027. Of these, 1536 were up-regulated, and 1761 were down-regulated (Fig. 2B). About 387 DEGs were identified in dataset GSE12654. Of these, 150 were up-regulated, and 237 were down-regulated (Fig. 2C). The three datasets were combined, and it was shown from the UMAP plots that prior to removing the batch effect, the samples of each dataset clustered together individually, indicating the presence of the batch effect. The samples of each dataset clustered and intertwined with each other after removing the batch effect, suggesting that the bathch effect was removed in a preferable manner (Fig. 2D, E). About 1392 DEGs were identified from the combined datasets. Of these, 667 were up-regulated, and 725 were down-regulated (Fig. 2F).

### Identification of DEGs between BD and MDD

**Fig. 3:**
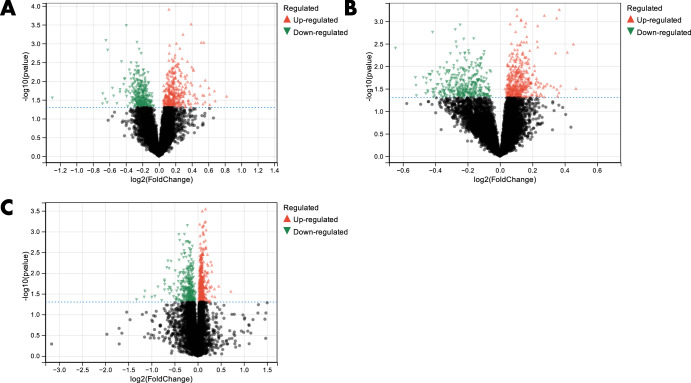
DEGs between BD and MDD. **A** volcano plot of DEGs in dataset GSE92538-GPL10526; **B** volcano plot of DEGs in dataset GSE92538-GPL17027; **C** volcano plot of DEGs in the combined dataset.

The Limma method was employed for the identification of about 650 DEGs in the dataset GSE92538-GPL10526. Of these, 346 were up-regulated, and 304 were down-regulated (Fig. 3A). About 779 DEGs were found in the dataset GSE92538-GPL17027. Of these, 431 were up-regulated, and 348 were down-regulated (Fig. 3B). About 802 DEGs were identified from the combined dataset. Of these, 478 were up-regulated, and 324 were down-regulated (Fig. 3C).

### Functional enrichment analysis of relevant candidate genes for BD to distinguish SC and MDD

**Fig. 4:**
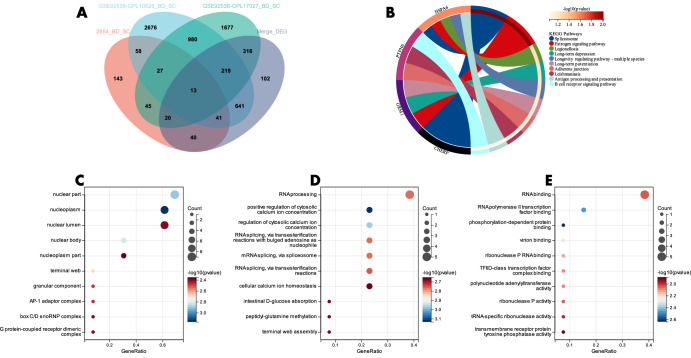
Functional enrichment analysis of relevant candidate genes distinguishing BD from SC. **A** Venn diagram of DEGs between bipolar disorder and schizophrenia in a single dataset versus DEGs in a combined dataset; **B** KEGG analysis of candidate genes; GO analysis of candidate genes for **C** cellular components (CC); **D** biological processes (BP); **E** molecular function (MF).

We cross-referenced the DEGs between BD and SC within a single dataset with those from the combined dataset, resulting in 13 candidate genes (Fig. 4A). These intersecting genes are considered capable of distinguishing BD and SC effectively in different datasets.The DEGs between BD and SC in the single dataset were crossed with those in the combined dataset to obtain 13 candidate genes (Fig. 4A). Functional enrichment analysis and KEGG analysis indicated the candidate genes to be mainly enriched in the “Spliceosome,” “Estrogen signaling pathway,” and “Legionellosis” pathways (Fig. 4B). GO evaluation depicted the candidate genes to be mainly located in the “nuclear part,” “nucleoplasm,” and “nuclear lumen “ with respect to cellular components (CC) (Fig. 4C). The main biological process (BP) of the candidate genes was “RNA processing” (Fig. 4D). Molecular function (MF) indicated “RNA binding” and “RNA polymerase II transcription factor binding” to the most vital items of candidate genes (Fig. 4E).

**Fig. 5:**
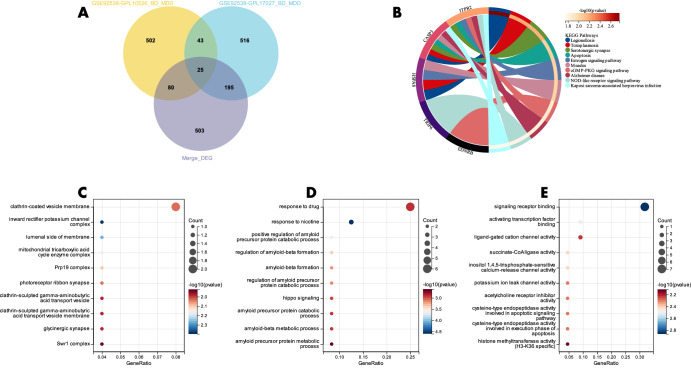
Functional enrichment analysis of relevant candidate genes distinguishing BD from MDD. **A** Venn diagram of DEGs between bipolar disorder and schizophrenia in the single dataset versus DEGs in the combined dataset; **B** KEGG analysis of candidate genes; GO analysis of candidate genes for **C** cellular components (CC); **D** biological processes (BP); **E** molecular function (MF).

Continuing, we conducted a cross-reference of DEGs between BD and MDD within an individual dataset with those from the merged dataset, resulting in 25 candidate genes (Fig. 5A). These intersecting genes are considered to effectively distinguish BD from MDD across different datasets.The DEGs between BD and MDD in the single dataset were crossed with those in the combined dataset to obtain 25 candidate genes (Fig. 5A). Functional enrichment analysis was carried out, and KEGG analysis exhibited the candidate genes to be mainly enriched in “Legionellosis,” “Toxoplasmosis,” and “Apoptosis “ immune pathways (Fig. 5B). GO evaluation indicated the candidate genes to be mainly located in the “clathrin-coated vesicle membrane” with respect to CC (Fig. 5C). The main biological processes (BP) of the candidate genes were “response to the drug” and “response to nicotine” (Fig. 5D). Molecular functions (MF) exhibited “signaling receptor binding,” “activating transcription factor binding,” and “ligand-gated cation channel activity” to be the most vital items of the candidate genes (Fig. 5E).

### Identification of BD to distinguish SC and MDD-related candidate genes by machine learning and PPI network construction

**Fig. 6:**
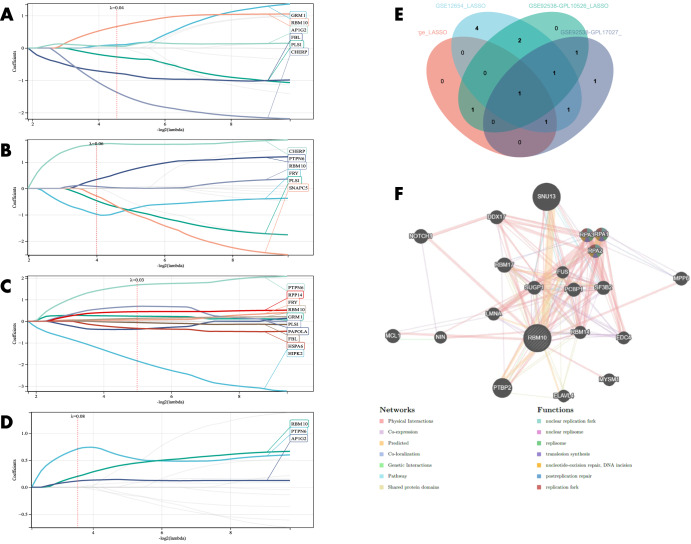
Candidate gene identification to distinguish BD from SC. **A–D** LASSO regression candidate gene identification (GSE92538-GPL10526, GSE92538-GPL17027, GSE12654, and combined datasets, respectively); **E** LASSO regression candidate gene Venn diagram; **F** PPI network construction of candidate genes.

**Fig. 7:**
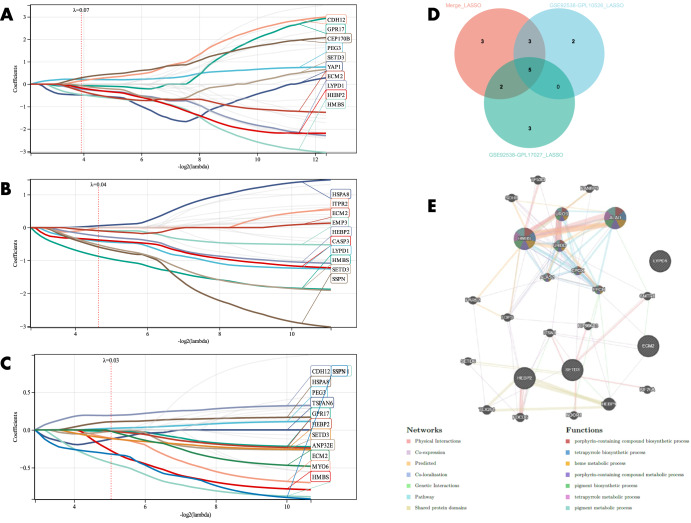
Candidate gene identification to distinguish BD from MDD. **A–C** LASSO regression candidate gene screening (GSE92538-GPL10526, GSE92538-GPL17027, and combined dataset, respectively); **D** LASSO regression candidate gene Venn diagram; **E** PPI network construction of candidate genes.

LASSO regression was applied for candidate gene identification to distinguish BD from SC. From the results, six potential candidate genes were identified in GSE92538-GPL10526 (Fig. 6A); six potential candidate genes were identified in GSE92538-GPL17027 (Fig. 6B); 10 potential candidate genes were identified in GSE12654 (Fig. 6C); and three potential candidate genes were identified in the combined dataset (Fig. 6D). Crossover was performed by Venn diagram, and RBM10 was obtained as a candidate gene to distinguish BD and SC (Fig. 6E), and through this RBM10, a PPI network was established, among which Physical Interactions made up 77.64%, and Co-expression made up 8.01%. These genes are mainly involved in nuclear replisome, replisome, and translesion synthesis (Fig. 6F). LASSO regression was then applied for candidate gene identification to distinguish BD from MDD. From the results, 10 potential candidate genes were identified in each of the GSE92538-GPL10526 (Fig. 7A), and GSE92538-GPL17027 datasets (Fig. 7B), and 13 potential candidate genes were identified in the combined dataset (Fig. 7C). Five candidate genes (LYPD1, HMBS, HEBP2, SETD3, and ECM2) capable of distinguishing BD from MDD were obtained by a crossover in the Venn diagram (Fig. 7D), and the PPI network was constructed using these five candidate genes, among which Physical Interactions made up 77.64%, and Co-expression made up 8.01%. These genes were mainly involved in the porphyrin-containing compound, tetrapyrrole, and heme metabolic biosynthetic processes (Fig. 7E).

### Diagnostic model validation

**Fig. 8:**
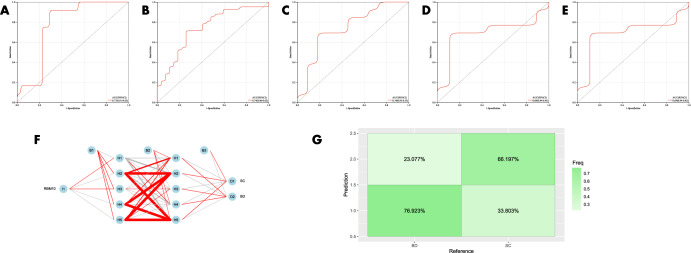
Validation of the diagnostic value of candidate genes for BD and SC. **A–E** ROC curves for different datasets (GSE92538-GPL10526, GSE92538-GPL17027, GSE12654, combined dataset, and GSE18312, respectively); **F**, **G** ANN validation of candidate genes.

The diagnostic value of the candidate genes (RBM10) distinguishing BD from SC was initially validated using ROC curves. The results of GSE92538-GPL10526 are exhibited in Fig. 8A (AUC 0.77, CI 0.91–0.63), GSE92538-GPL17027 in Fig. 8B (AUC 0.74, CI 0.86–0.62), GSE12654 in Fig. 8C (AUC 0.74, CI 0.95–0.53), and the combined dataset in Fig. 8D (AUC 0.74, CI 0.95–0.53). In order to validate it, the diagnostic model was also put into the peripheral blood validation group (GSE18312), and the findings exhibited a good diagnostic significance (AUC 0.69, CI 0.94–0.45) (Fig. 8E). The candidate genes were employed for the construction of ANN, and the outcomes showed that the candidate genes were capable of distinguishing between fine BD and SC, and the accuracy could reach 76.923% (Fig. 8F, G). The expression profile analysis of candidate genes was also evaluated, and in all datasets, RBM10 expression was significantly different (*P* < 0.05) in BD and SC (Fig. 10A–D), and SC expression was higher than BD in all cases.

**Fig. 9:**
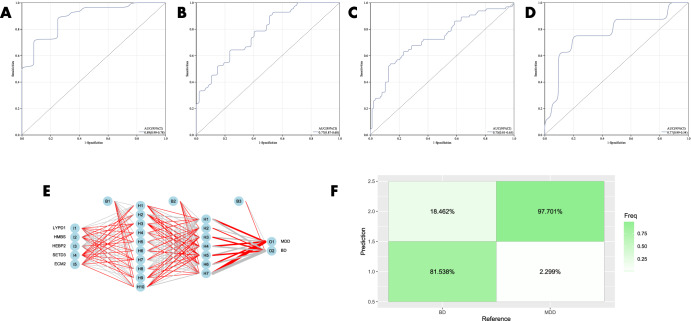
Validation of the diagnostic value of candidate genes for BD and MDD. **A**–**D** ROC curves for different datasets (GSE92538-GPL10526, GSE92538-GPL17027, combined dataset, and GSE39653, respectively); **E**, **F** ANN validation of candidate genes.

**Fig. 10:**
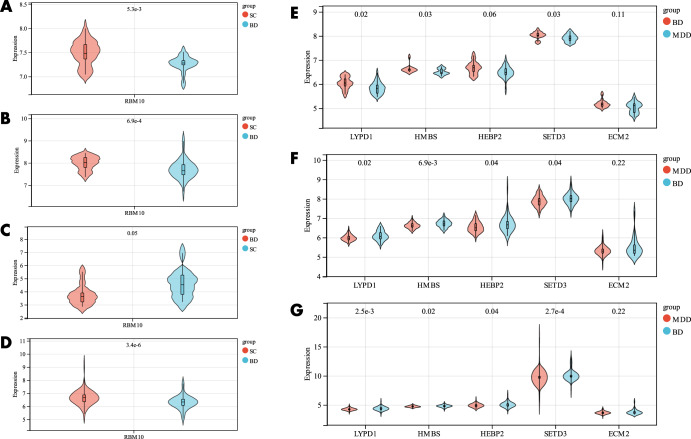
The expression profile analysis of candidate genes. **A**–**D** differential expression profiling of candidate genes distinguishing BD and SC (GSE92538-GPL10526, GSE92538-GPL17027, GSE12654, and combined datasets, respectively); **E**–**G** differential expression profiling of candidate genes distinguishing BD and MDD (GSE92538- GPL10526, GSE92538-GPL17027, and the combined dataset).

The diagnostic value of the candidate genes(LYPD1,HMBS,HEBP2,SETD3 and ECM2)distinguishing BD from MDD was then evaluated using ROC curves, which were (AUC 0.89, CI 0.99–0.78) in GSE92538-GPL10526 (Fig. 9A); (AUC 0.77, CI 0.87–0.68) in GSE92538-GPL17027 (Fig. 9B); and (AUC 0.77, CI 0.87–0.68) in the combined dataset was (AUC 0.73, CI 0.81–0.65) (Fig. 9C). In order to validate it, the diagnostic model was also put into the peripheral blood validation group (GSE39653) for validation, and the outcomes exhibited a good diagnostic significance (AUC 0.77, CI 0.99–0.54) (Fig. 9D). Five candidate genes were employed for the construction of the ANN, and the findings showed that the candidate genes were able to distinguish well between BD and MDD, and the accuracy could reach 81.538% (Fig. 9E, F). And there were significant differences in LYPD1, HMBS, and SETD3 expression in BD and MDD in all datasets (*P* < 0.05) (Fig. 10E–G),and BD expression was higher than MDD in all cases.

### Immune cell infiltration analysis

**Fig. 11:**
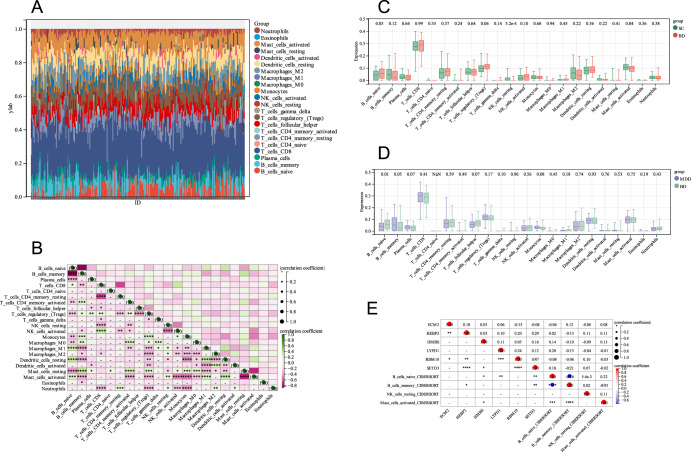
CIBERSORT analysis of 22 immune cells. **A** relative percentage of 22 immune cells in each sample; **B** correlation among 22 immune cells; **C** difference in immune infiltration between BD and SC; **D** difference in immune infiltration between BD and MDD; **E** correlation among 6 genes and 4 immune cells.

In this research, the proportion of 22 immune cells in BD, SC, and MDD in the combined groups was estimated by the CIBERSORT algorithm (Fig. 11A, B). A comparison of immune cell infiltration among BD and SC was carried out in box plots (Fig. 11C), and the results showed a significant difference in Naive B, Resting NK, and Activated Mast Cells between the two groups (*P* < 0.05). A comparison of immune cell infiltration was also carried out between BD and MDD (Fig. 11D), and the results showed significant differences in Naive B and Memory B cells among the two subsets (*P* < 0.05). Furthermore, we conducted correlation analysis between six target genes (RBM10, LYPD1, HMBS, HEBP2, SETD3, and ECM2) and significantly different immune cells, and the results showed that HMBS and B_ Cells_ Naive_ CIBERSORT and Mast_ Cells_ Activated_ Significant correlation between CIBERSORT, HEBP2 and B_ Cells_ Memory_ Significant correlation between CIBERSORT, LYPD1 and B_ Cells_ Naive_ Significant correlation between CIBERSORT, SETD3 and B_ Cells_ Naive_ CIBERSORT and B_ Cells_ Memory_ CIBERSORT is significantly correlated. (Fig. 11E)

## Discussion

For the purpose of differentiating BD from SC and MDD, models were obtained by bioinformatics analysis and machine learning screening. The differentiation between BD and SC by RBM10 proved effective. Expression of RBM10 appeared greater in the SC group as opposed to the BD group. According to the KEGG analysis, a correlation between BD and SC may be due to the existence of spliceosome. Immune infiltration analysis was also carried out, which showed a prominent variation among the two groups for Naive B, Resting NK, and Activated Mast Cells (*P* < 0.05). In addition, immune infiltration analysis was performed, and the findings indicated a substantial variation among Naive B and Memory B cells in BD and MDD (*P* < 0.05).

RBM10 is a protein-coding gene linked to the hnRNP protein and may be involved in regulating selective splicing. Very few existing studies focused on the correlation between RBM10 and SC or BD, but it was observed that disruption at the level of selective splicing isoforms rather than the level of gene expression is a major source of pathological effects in psychiatric and neurological disorders^20^. The results of Michael J. Gandal et al. showed that by genotyping and RNA sequencing in brain samples from 1695 subjects with autism, SC, BD, and controls, more than 25% of the transcriptome exhibited differential splicing or expression, with changes in isoform levels capturing the greatest disease effects and genetic enrichment, and co-expression networks showing disease-specific neuronal mutation^21^.

LYPD1 (Lynx2) is a nAChR regulator that is expressed in postmitotic central and peripheral neurons in embryonic and postnatal mice^22^ and regulates α4β2-nAChRs^23^. According to Ayse B Tekinay et al., deletion of Lynx2 leads to elevated anxiety-like behavior, suggesting that LYNX2 binds and regulates neuronal nicotinic acetylcholine receptors and that deletion of Lynx2 alters the action of nicotine on prefrontal cortical glutamatergic signaling^23^.

Numerous research has revealed the importance of vascular endothelial growth factor (VEGF) in antidepressants^24^, which vitally mediates the neurogenic and behavioral actions of several antidepressants^25^. The neurogenic and neuroprotective actions of VEGF are capable of influencing hippocampal-dependent processes, including learning and memory^26^. In addition, SETD3 interacts with FoxM1 at the VEGF promoter, and it is capable of transcriptionally regulating the expression of VEGF. It was observed that SETD3 knockdown alleviates depressive symptoms in post-stroke depression in rats based on a study by Yun Feng et al. on murine post-stroke depression^27^.

This research has several limitations^1^; While being useful in this investigation, the diagnostic prediction model did not receive further validation through experimentation^2^. Due to the lack of corresponding clinical correlation studies, analysis of the model concerning clinical information could not be conducted.

## Conclusion

In this study, RBM10 was found as a candidate gene to distinguish BD from SC and LYPD1, HMBS, HEBP2, SETD3, and ECM2 as five candidate genes to distinguish BD from MDD. The results obtained from the ANN network showed that the candidate genes could better distinguish BD from SC and MDD (76.923% and 81.538%).

### Supplementary information

Supplementary Table

## Data Availability

The datasets generated and analysed during the current study are available in the [GEO] repository, [https://www.ncbi.nlm.nih.gov/geo/].
